# Whisky Doctors

**Published:** 1876-06

**Authors:** 


### WHISKY-DOCTORS.

A CONSIDERABLE number of persons applying to us for the treatment of throat and lung affections, in answer to the inquiry, "What have you been doing for your ailment," reply, "Using Bourbon whisky:" nine times in ten adding, "It seemed to do good for a while, but has not effected any permanent benefit." Very many persons troubled with a tickling in the throat or annoying hawking or hemming, as a consequence of a disorder of the stomach, evidenced by a changeable or indifferent appetite, bad taste in the mouth of mornings, cold feet, or general chilliness, have been "advised to take a little whisky at meals." The very fact of their seeking further counsel after trying the whisky treatment, is conclusive of its inefficiency. In nearly all the cases, especially from New-England, the practice has been adopted by the "advice of the family physician." One of the best American surgeons known to us, is the most inveterate liquor-drinker.

